# Structure-based multi-pocket virtual screening identifies FDA-approved candidate compounds targeting MPXV thymidylate kinase

**DOI:** 10.3389/fcimb.2026.1743154

**Published:** 2026-09-14

**Authors:** Hanwen Lu, Xiaodong Li, Zhanxiang Wang

**Affiliations:** 1 Department of Neurosurgery and Department of Neuroscience, Fujian Key Laboratory of Brain Tumors Diagnosis and Precision Treatment, Xiamen Key Laboratory of Brain Center, the First Affiliated Hospital of Xiamen University, School of Medicine, Xiamen University, Xiamen, China; 2 College of Integrative Medicine, Fujian University of Traditional Chinese Medicine, Fuzhou, China; 3 Institute of Clinical Medicine, The First Affiliated Hospital of Xiamen University, Xiamen University, Xiamen, Fujian, China; 4 Xiamen Key Laboratory of Brain Center, The First Affiliated Hospital of Xiamen University, Xiamen University, Xiamen, Fujian, China

**Keywords:** drug repurposing, molecular dynamics, monkeypox virus, thymidylate kinase, virtual screening

## Abstract

**Background:**

Mpox, caused by monkeypox virus (MPXV), has emerged as a growing global health concern, yet effective targeted antiviral therapies remain limited. Thymidylate kinase (TMK), an essential enzyme required for viral DNA replication, represents a potential therapeutic target.

**Methods:**

We established a structure-based drug repurposing workflow integrating multi-pocket virtual screening and molecular dynamics (MD) simulations. A curated library of FDA-approved compounds was screened across five predicted TMK binding pockets, followed by consensus ranking based on cross-pocket recurrence and docking scores. Top candidates were further evaluated using multiple independent MD simulations to assess binding stability and conformational dynamics.

**Results:**

Phylogenetic and structural analyses indicated that MPXV TMK is highly conserved among orthopoxviruses while structurally distinct from the human homolog. Virtual screening identified several prioritized candidates, among which capmatinib (DB11791), nebivolol (DB04861), and tucatinib (DB11652) consistently ranked highly across multiple pockets. MD simulations showed that all systems approached relatively stable conformational regimes after the initial phase of the simulations. Notably, DB11791 exhibited lower ligand RMSD and more stable pocket retention across independent simulations. Free energy landscape analysis further suggested that DB11791 adopted a more confined low-energy conformational state compared to other candidates.

**Conclusion:**

These findings suggest that DB11791 may represent a promising TMK-targeting candidate. More broadly, the combined multi-pocket consensus screening and dynamic evaluation strategy provides a computational framework for antiviral drug repurposing. Further experimental validation is required to confirm the antiviral activity of these candidates.

## Graphical Abstract

**Figure:**
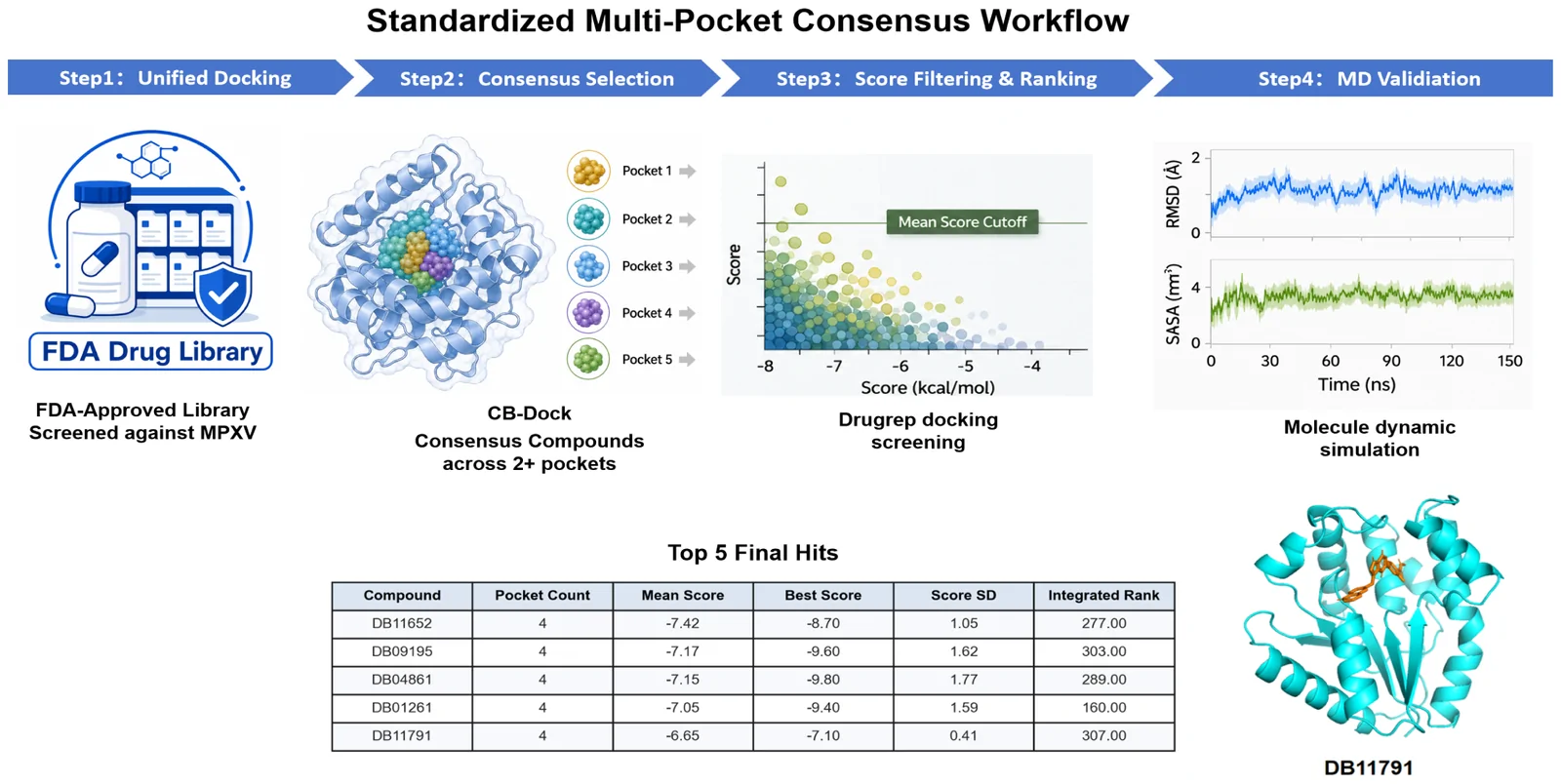

## 1 Introduction

Presently, the outbreak of mpox poses a new threat and challenge to our society (Ahmed et al., 2022; Hraib et al., 2022; World Health Organization, 2022; Global strategic preparedness and response plan launched by WHO to contain mpox outbreak, 2024). On July 23, 2022, the World Health Organization declared the ongoing MPX epidemic as a Public Health Emergency of International Concern (PHEIC) (McQuiston et al., 2023). The 2022 outbreak represented the first sustained global outbreak of mpox with widespread human-to-human transmission and the largest reported to date since the disease was first recognized in humans in 1970 (Gessain et al., 2022). Historically, mpox outbreaks outside Africa were rare and largely sporadic before 2022; in contrast, the 2022 outbreak rapidly expanded worldwide, with more than 55, 000 confirmed cases reported within a few months (Laurenson-Schafer et al., 2023).

MPXV was first identified by virologists Preben von Magnus and his colleagues in 1959 during the investigation of two smallpox-like disease outbreaks in crab-eating macaques. The first human case of mpox was reported in 1970 (Von Magnus et al., 1959; Breman et al., 1980). In the current outbreak, human-to-human transmission has predominated, with close physical contact, including sexual contact, accounting for a large proportion of reported cases. Although mpox is a self-limiting disease typically lasting 2 to 4 weeks, reports of severe symptoms and complications include secondary infections, rectal inflammation, tonsillitis, bronchopneumonia, sepsis, encephalitis, and the prognosis of mpox depends on various factors such as initial health status, comorbidities, and complications (Tang and Zhang, 2023). Patients with compromised immune systems are at higher risk of severe mpox and poor clinical outcomes. No universally established antiviral regimen has been approved specifically for mpox, and treatment options remain limited (Siami et al., 2023).

Monkeypox virus (MPXV) is an enveloped double-stranded DNA virus belonging to the genus Orthopoxvirus, subfamily Chordopoxvirinae, and family Poxviridae (Shchelkunov et al., 2001; Ferrareze et al., 2023; McInnes et al., 2023; Black et al., 2026). MPXV harbors a large double-stranded DNA genome encoding numerous viral proteins involved in replication, virion assembly, and host interaction (Resch et al., 2007; Manes et al., 2008; Schmidt et al., 2012; Gigante et al., 2022; Isidro et al., 2022; Luna et al., 2022; Minasov et al., 2022). Its replication depends on a coordinated set of viral proteins involved in nucleotide metabolism, DNA synthesis, and genome processing, making replication-associated enzymes attractive candidates for antiviral intervention (Prichard and Kern, 2012; Moss, 2013; Li et al., 2023). The natural reservoir of MPXV remains uncertain, but various small mammals, particularly African rodents, are thought to contribute to viral maintenance in nature (Alakunle et al., 2020; Gessain et al., 2022; Hutson et al., 2015; Falendysz et al., 2017; Riutord-Fe et al., 2026). Among these, thymidylate kinase (TMK) is considered a potential antiviral target due to its essential role in nucleotide metabolism and viral DNA replication.

In light of these challenges, the present study aims to systematically evaluate MPXV TMK as a therapeutic target and to establish a multi-dimensional computational pipeline for drug repurposing. Unlike previous studies that mainly relied on single-pocket docking or small-scale libraries, we introduced a multi-pocket consensus screening strategy that integrates structure-based docking, ADMET prediction, and molecular dynamics simulations. This strategy is designed to improve candidate prioritization by considering multiple binding cavities simultaneously and to identify compounds with consistent binding features across different structural contexts. Furthermore, by focusing on FDA-approved drugs, our approach provides a translationally relevant framework that may accelerate the transition from computational prediction to experimental validation. Through this work, we highlight TMK as a biologically relevant and insufficiently validated antiviral target, but also propose a generalizable workflow that could be extended to other viral pathogens in future outbreaks.

## 2 Methods

### 2.1 Epidemiological and clinical data collection and analysis

Global mpox case data were collected from the World Health Organization (WHO), the U.S. Centers for Disease Control and Prevention (CDC), and publicly accessible national public health databases, with data retrieval completed on December 30, 2024. Case distribution and regional epidemiological trends were organized in Microsoft Excel and visualized in R (v4.2.0). Clinical trial information was retrieved from ClinicalTrials.gov and the WHO International Clinical Trials Registry Platform (ICTRP) using the keywords “Monkeypox, “ “MPXV, “ “antiviral, “ and “vaccine.” Information on reported antiviral targets and relevant signaling pathways was collected from the KEGG and Reactome databases and further integrated with published literature. Key events in the mpox virus life cycle and host-related signaling pathways were summarized on the basis of previous orthopoxvirus studies and illustrated using professional scientific drawing software.

### 2.2 Viral structure and target analysis

The genome sequence of monkeypox virus (MPXV) was obtained from the NCBI GenBank database. Schematic illustrations of viral structure and genome organization were generated based on published cryo-electron microscopy and genome annotation studies. TMK protein sequences from representative orthopoxviruses were aligned using ESPript3.0 (Robert and Gouet, 2014), and phylogenetic analysis and sequence-conservation visualization were performed using the NGPhylogeny.fr web platform (Lemoine et al., 2019). The three-dimensional structure of MPXV TMK was predicted using AlphaFold2, followed by structural comparison with related orthopoxvirus TMKs and human thymidylate kinase. Structural visualization, inspection of the catalytic region, and preliminary binding-site analysis were performed in PyMOL.

### 2.3 Database construction

Structure-based screening was performed using an FDA-approved drug library compiled from public drug repositories and standardized for virtual screening (O’Boyle et al., 2011; Wishart et al., 2018). Chemical structures were collected, curated, and deduplicated, and a final set of 2, 315 FDA-approved compounds was retained for subsequent analysis. The ligand structures were converted into PDBQT format using Open Babel. This library was used as the sole screening set in the revised workflow to reduce chemical-space bias arising from mixed-source compound collections and to better align the study with the objective of antiviral drug repurposing.

### 2.4 Protein preparation and virtual screening

The DrugRep platform was used for structure-based virtual screening (Gan et al., 2023). The MPXV TMK structure was predicted by AlphaFold2, and the protein was prepared by removing water molecules, adding hydrogen atoms, and assigning Gasteiger charges using AutoDock Tools (Gasteiger and Marsili, 1980; Morris et al., 2009). Because no experimental MPXV TMK-ligand complex structure is available, TYD from the homologous poxvirus TMK crystal structure 2V54 (Caillat et al., 2008) was used as a reference. Five putative ligandable pockets were identified using the native cavity-recognition algorithm in DrugRep and cross-validated with CB-Dock, and their definitions are provided in **Supplementary Table 1**.

### 2.5 Molecular docking

Virtual screening was performed using the DrugRep platform with its integrated AutoDock Vina engine. The FDA-approved drug library was screened independently against each of the five predefined pockets of MPXV TMK. After complete docking of the full FDA-approved library to each pocket, the top 200 ranked compounds from each pocket were retained for downstream cross-pocket comparison and consensus prioritization. This standardized cutoff ensured that the intersection analysis was performed under a unified candidate depth across all pockets. Consensus candidates were ranked by integrating pocket recurrence and docking performance, including pocket coverage, mean docking score, best docking score, and score variability. Additional interaction-based prioritization was performed using interaction fingerprint (IFP) similarity clustering and residue-level interaction pattern analysis. To validate the docking workflow, the original co-crystallized ligand TYD from the poxvirus TMK crystal structure 2V54 was used as a reference ligand for redocking (Trott and Olson, 2010). TYD was re-docked into the corresponding binding region, and the best-ranked pose was compared with the crystallographic pose by structural superposition to assess the ability of the docking protocol to reproduce the experimentally observed binding mode. The redocking result was further used as a positive-reference control for subsequent molecular dynamics simulations. CB-Dock was used as an auxiliary pocket-recognition and blind-docking tool to support cavity identification and cross-check the plausibility of the selected binding region (Liu et al., 2020). Final docking scores used for candidate prioritization were obtained from AutoDock Vina.

### 2.6 ADMET prediction for candidate compounds

ADMET properties of the top-ranked candidate compounds were predicted using admetSAR3.0 (Gu et al., 2024). The evaluated parameters included human intestinal absorption (HIA), blood–brain barrier (BBB) penetration, P-glycoprotein (P-gp) substrate tendency, human and rat liver microsomal stability (HLM and RLM), drug-induced liver injury (DILI), and hERG-related liability. These predictions were used as supportive evidence for relative candidate prioritization rather than as definitive clinical safety conclusions.

### 2.7 Molecular dynamics simulations

Molecular dynamics (MD) simulations were carried out for prioritized TMK–ligand complexes, including the reference ligand TYD and selected candidate compounds. Ligand parameters were generated with AmberTools and converted using ACPYPE, and all systems were prepared in GROMACS 2022. After solvation, ion neutralization, energy minimization, and NVT/NPT equilibration, three independent 150 ns production runs were performed for each complex. Trajectories were analyzed using RMSD, hydrogen-bond persistence, radius of gyration (Rg), solvent-accessible surface area (SASA), PCA, and FEL analyses to assess structural stability and dynamic binding behavior.

### 2.8 Interaction fingerprint analysis

Interaction fingerprint (IFP) analysis was performed using custom scripts written in Python 3.9 to compare the binding modes of candidate compounds with the target protein. Representative protein–ligand complex conformations were used as input, and key non-covalent interactions within the binding pocket, including hydrogen bonds, hydrophobic contacts, aromatic stacking, cation-π interactions, and electrostatic interactions, were identified and encoded into standardized fingerprint matrices. These matrices were then used to compare interaction patterns among different compounds. Data processing and organization were carried out using NumPy and Pandas, visualization was performed with Matplotlib, and PCA and hierarchical clustering were implemented using scikit-learn and SciPy, respectively.

### 2.9 Heatmap visualization

Heatmaps were generated using custom scripts implemented in Python 3.9. Quantitative results, including docking scores, interaction fingerprint features, molecular dynamics parameters, and other numerical descriptors, were organized into a two-dimensional matrix in the format of “compound × feature.” The data were standardized when necessary, for example by Z-score normalization, to improve comparability among variables. Pandas and NumPy were used for data preprocessing, Matplotlib for heatmap visualization, and SciPy and scikit-learn for hierarchical clustering and related analyses when required.

## 3 Results

### 3.1 Therapeutic target landscape of Mpox with a focus on TMK

To characterize the global burden of mpox and its implications for antiviral drug development, we first analyzed its epidemiological distribution and transmission patterns (**Figures 1A–C**). Reported mpox cases were predominantly concentrated in the Americas and Europe, while Africa, the historically endemic region, accounted for 21.3% of reported cases. This distribution indicates that mpox has expanded beyond localized endemic transmission and has become a global public health concern. Human-to-human transmission now dominates, with close physical contact, particularly sexual contact within affected transmission networks, accounting for a large proportion of reported cases.

**Figure 1:**
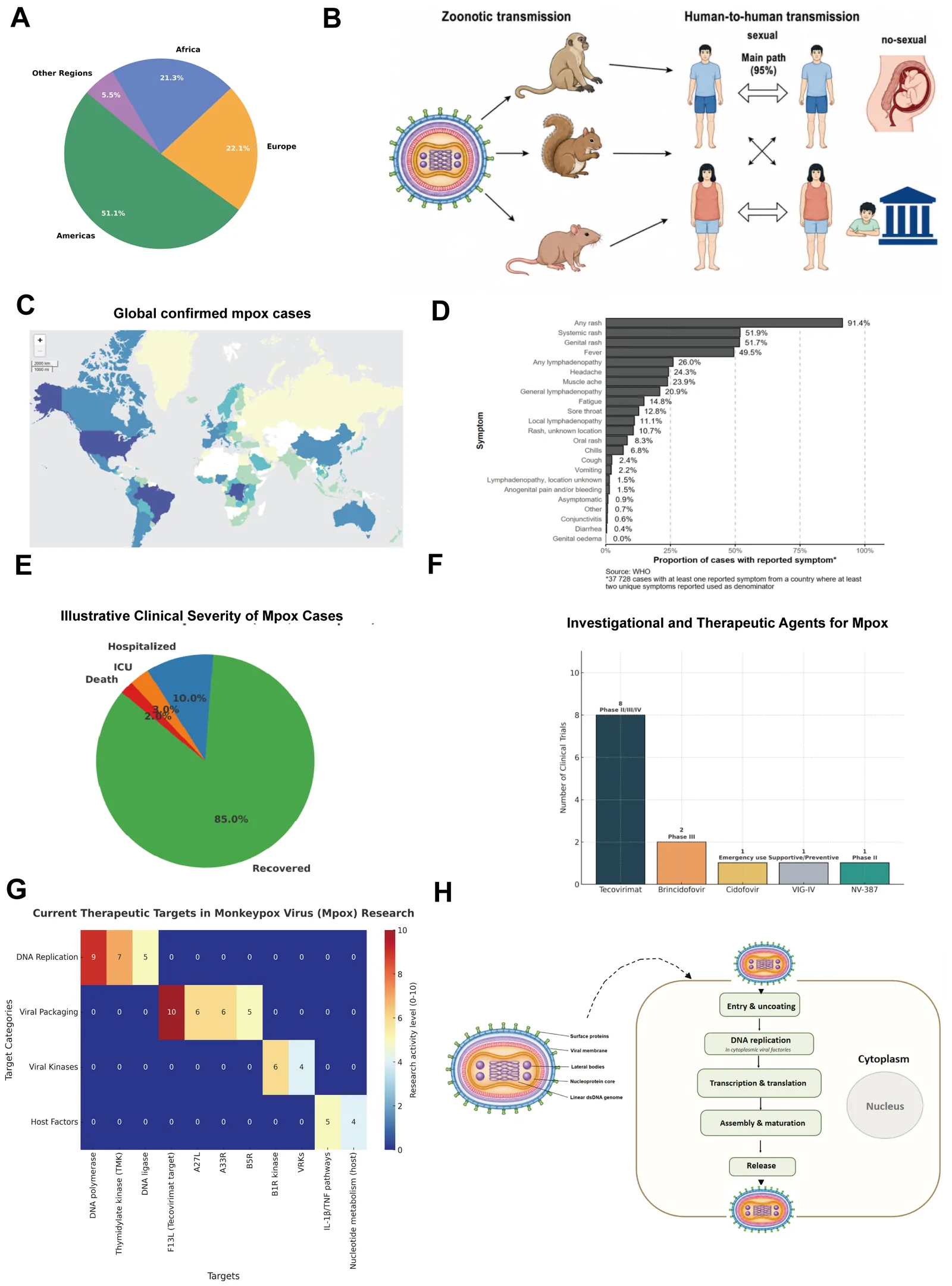
Epidemiology, clinical features, and therapeutic targets of Mpox. **(A)** Regional distribution of reported mpox cases worldwide, showing the relative proportions across the Americas, Europe, Africa, and other regions. **(B)** Major MPXV transmission routes, including zoonotic transmission and person-to-person transmission through close physical and other non-sexual contact. **(C)** Global map of reported mpox cases based on publicly available surveillance data from WHO and CDC sources. **(D)** Summary of the major clinical manifestations of mpox, highlighting the frequency of commonly reported symptoms, including rash, lymphadenopathy, and systemic manifestations. **(E)** Illustrative summary of clinical severity and outcomes in mpox cases, including recovery, hospitalization, ICU admission, and death. The categories are not mutually exclusive, and the percentages should not be interpreted as pooled global estimates. **(F)** Current landscape of antiviral agents and clinical development against mpox, including representative compounds such as tecovirimat, brincidofovir, cidofovir, VIG-IV, and NV-387, categorized according to their development or trial status. **(G)** Overview of current therapeutic targets investigated in mpox research, grouped into major functional categories such as viral DNA replication, viral packaging, viral kinases, and host-related factors. Signal intensity reflects the relative degree of research attention. **(H)** Schematic illustration of MPXV structure and key virus-related biological processes, highlighting representative therapeutic targets involved in viral replication and host–virus interactions, including TMK and RNA polymerase.

We next examined the clinical manifestations and disease outcomes associated with mpox infection (**Figures 1D, E**). The most frequently reported symptoms included rash, lymphadenopathy, and systemic manifestations, whereas fever, headache, and myalgia occurred less consistently. Among reported cases, approximately 10% required hospitalization, 2% required intensive care, and about 1% resulted in mortality. These findings underscore that mpox can pose significant risks, particularly for vulnerable populations such as immunocompromised individuals.

Given the ongoing global spread and clinical burden, we further evaluated the current landscape of antiviral drug development (**Figure 1F**). Tecovirimat represents the most advanced therapeutic candidate and is currently undergoing phase III clinical evaluation, while brincidofovir and cidofovir remain in earlier stages. Several additional agents, including VIG-IV and NV-387, are still under exploratory investigation. Overall, the current therapeutic pipeline remains limited, emphasizing the urgent need for novel and targeted antiviral strategies.

To identify potential molecular targets, we systematically analyzed key processes involved in mpox virus infection and replication (**Figures 1G, H**). Current research has focused on viral DNA replication, genome packaging, viral kinases, and host-related pathways, with replication-associated proteins receiving the most attention. Among these, TMK, a critical enzyme involved in nucleotide metabolism and DNA synthesis, TMK emerged as a potential antiviral target. TMK plays an essential role in viral genome replication and exhibits structural divergence from its human homolog, suggesting the potential for selective targeting. Collectively, these results highlight the global spread, clinical relevance, and limited therapeutic options for mpox, while identifying TMK as a rational and promising target for antiviral drug discovery.

3.2 Conserved Core and Host-Specific Divergence of MPXV TMK Reveal Opportunities for Selective Drug Targeting.

To characterize the molecular features of MPXV TMK and evaluate its potential as a therapeutic target, we performed a multi-level analysis integrating genomic localization, evolutionary relationships, structural organization, and sequence conservation. At the genomic level, the MPXV genome consists of inverted terminal repeats (ITRs), a central conserved region, and variable terminal regions. Key replication-related proteins, including DNA polymerase, TMK, and RNA polymerase, are all encoded within the conserved core region (**Figure 2A**). When considered alongside the distribution of functional targets across the viral life cycle, multiple intervention points can be identified, spanning DNA replication, transcription, assembly, and release (**Figure 2B**). Among these processes, TMK is directly involved in nucleotide metabolism and viral genome replication, highlighting its central role in maintaining viral replication.

**Figure 2:**
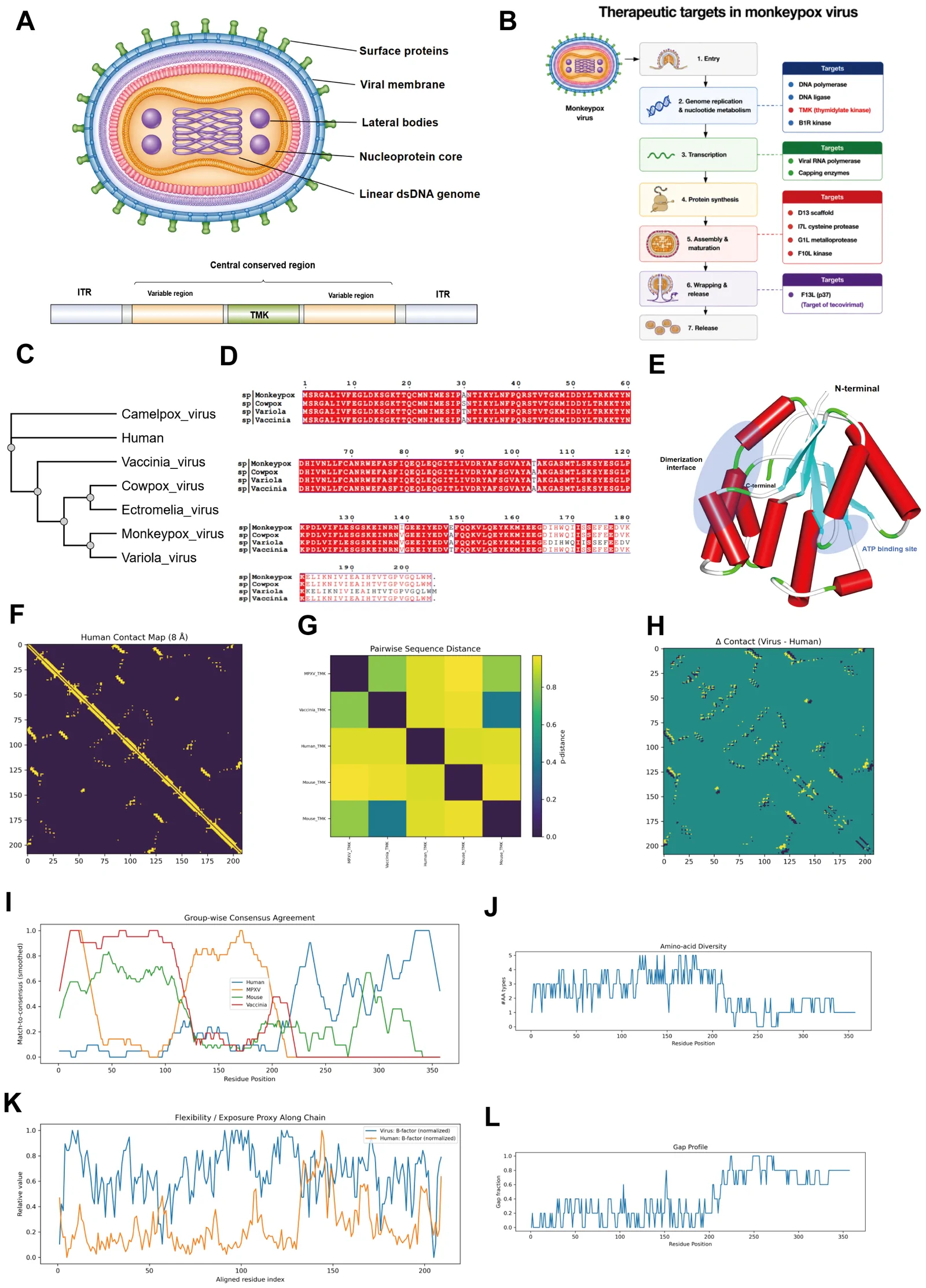
Structural and evolutionary features of TMK in Mpox. **(A)** Schematic diagram of the Mpox particle and genome organization. The genome consists of inverted terminal repeats (ITRs), a central conserved region, and variable regions; DNA polymerase, TMK, and RNA polymerase are highlighted as essential replication genes. **(B)** Therapeutic targets in the MPXV life cycle, including viral DNA replication, transcription, assembly and maturation, and wrapping/egress. TMK is emphasized as a key enzyme in DNA replication. **(C)** Phylogenetic relationship of TMK proteins, showing clustering of Mpox TMK with other orthopoxviruses and their separation from TMKs of other viral families. **(D)** Multiple sequence alignment of TMK proteins from Mpox and representative orthopoxviruses, highlighting conserved amino acid residues. **(E)** Predicted three-dimensional structure of MPXV TMK, showing secondary structural elements (α-helices in blue and β-sheets in yellow), key catalytic residues (Lys21, Asp95, and Glu142; red sticks), the ATP-binding pocket (orange), the TMP-binding site (green), and the dimerization interface. **(F)** Human TMK contact map. Pairwise residue–residue interactions within human TMK are shown, illustrating its structural organization. **(G)** Pairwise sequence distance matrix. MPXV TMK is compared with TMKs from related orthopoxviruses, with lower distance values indicating higher conservation. **(H)** Contact-map differences between viral and human TMKs. Subtraction analysis highlights residues with divergent contact patterns, reflecting structural differences between viral and human TMKs. **(I)** Group-wise consensus agreement profiles across TMK homologs. **(J)** Per-residue amino acid diversity across TMK homologs. **(K)** Normalized flexibility and solvent-exposure proxy profiles along the aligned sequence for viral and human TMKs. **(L)** Alignment gap-frequency profile across residue positions.

Phylogenetic analysis further supports the relevance of TMK as a conserved viral component. MPXV TMK clusters closely with TMKs from variola virus, vaccinia virus, and cowpox virus, while remaining clearly separated from homologs in other viral families such as herpesviruses and parvoviruses (**Figure 2C**). Multiple sequence alignment revealed that several key residues, including those associated with catalytic activity, are highly conserved across orthopoxviruses (**Figure 2D**), indicating strong evolutionary constraints within this genus.

Structural analysis showed that TMK adopts a typical α/β fold, with both the ATP-binding site and the TMP-binding pocket located within the central region of the protein (**Figure 2E**). Catalytic residues, including Lys21, Asp95, and Glu142, form the core of the active site and define the primary regions for ligand interaction. To assess potential differences between viral and host enzymes, contact map and sequence distance analyses were performed. These analyses revealed notable divergence in residue interaction patterns between MPXV TMK and the human homolog, particularly within the binding pocket region (**Figures 2F–H**). Such differences suggest that structural variations in this region may be exploited to achieve selective inhibition of the viral enzyme.

Sequence-based analyses further highlighted the distribution of conservation and variability within TMK (**Figures 2I–L**). While high sequence consistency was observed within the orthopoxvirus genus, clear divergence was evident when compared with the human homolog. Amino acid diversity and gap distribution analyses indicated that peripheral regions exhibit higher variability, whereas the catalytic core remains highly conserved. Flexibility and solvent accessibility analyses showed that surface loops display greater mobility, whereas the catalytic pocket is relatively rigid, providing a structurally favorable environment for ligand binding.

### 3.3 Structure-guided virtual screening reveals a multi-pocket inhibitor landscape for MPXV TMK

After identifying MPXV TMK as a potential antiviral target, we performed structure-based virtual screening to explore its ligand-binding landscape. A screening library was assembled and its functional composition was examined. The compounds covered a broad range of pharmacological categories and biological pathways, including neuronal signaling, metabolic processes, transmembrane transport, and immune and inflammatory regulation, as well as subsets related to DNA damage and repair, endocrine regulation, and GPCR signaling (**Figures 3A, C****).** Target-based classification showed that the library included antibacterial agents, antibiotics, adrenergic receptor modulators, autophagy-related compounds, and immune-associated molecules, together with additional classes involving apoptosis, acetylcholine receptors, serotonin receptors, nucleic acid synthesis, cyclooxygenases, histamine receptors, dopamine receptors, and calcium channels (**Figures 3B, D**). This distribution indicates that the screening set provides substantial chemical and pharmacological diversity, supporting its suitability for TMK-oriented screening.

**Figure 3:**
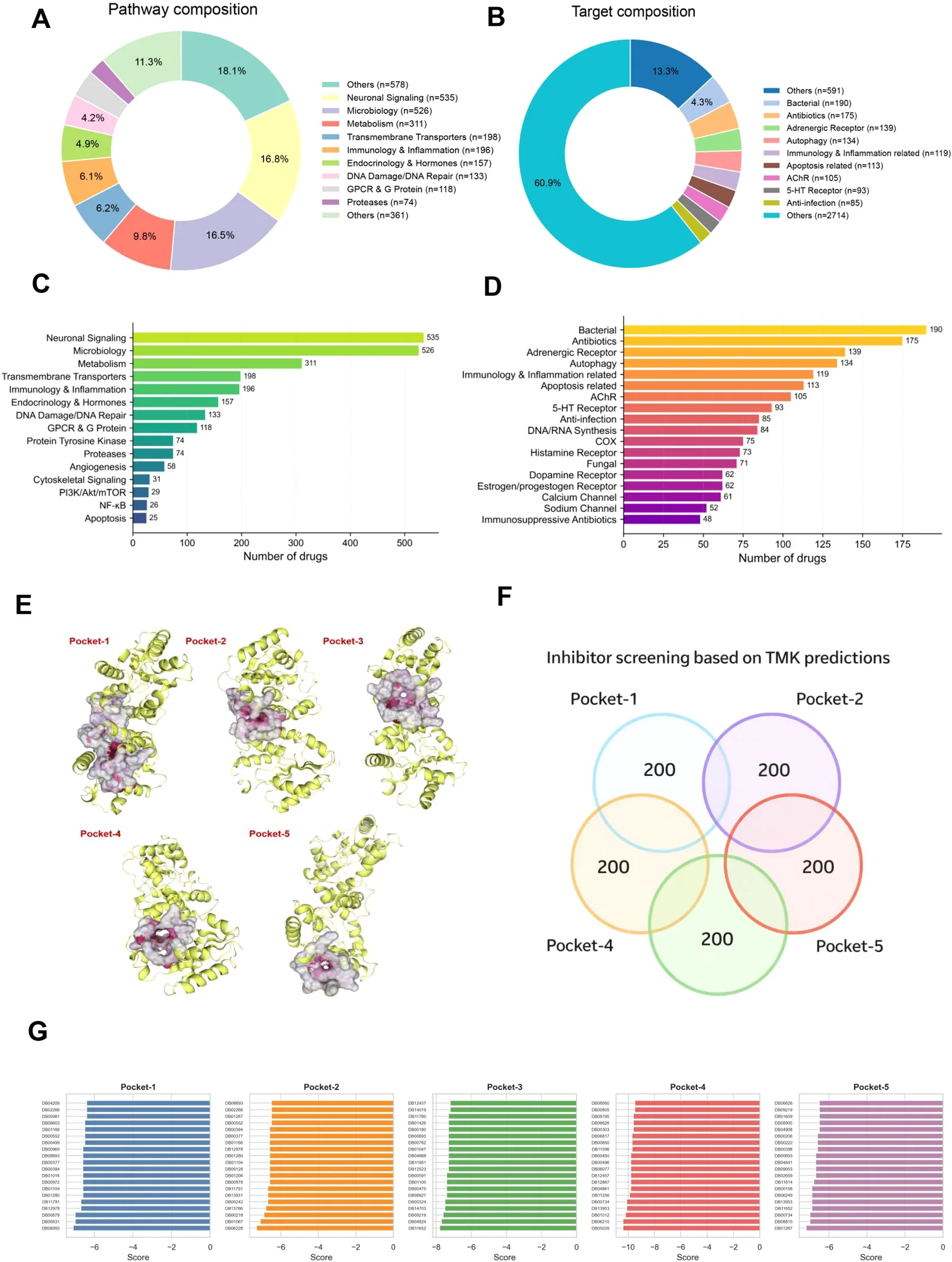
Structure-guided virtual screening framework for Mpox TMK based on a five-pocket strategy. **(A)** Donut chart showing the pathway composition of the screening library. The library covered diverse functional categories, including neuronal signaling, microbiology-related pathways, metabolism, transmembrane transporters, immunology and inflammation, endocrinology and hormones, DNA damage/repair, GPCR and G protein signaling, proteases, and others. **(B)** Donut chart showing the target composition of the screening library. The compounds were mainly classified into antibacterial agents, antibiotics, adrenergic receptor-related molecules, autophagy-related compounds, immunology and inflammation-related agents, and other target classes, including AChR, 5-HT receptor, DNA/RNA synthesis, COX, histamine receptor, dopamine receptor, calcium channel, and others. **(C)** Bar plot summarizing the number of compounds in each pathway-related category of the screening library. **(D)** Bar plot summarizing the number of compounds in each target-related category of the screening library. **(E)** Structural representation of Mpox TMK showing the five predicted ligand-binding pockets (Pocket-1 to Pocket-5) used for independent docking analyses. The pockets are distributed around the protein surface and provide multiple potential ligandable regions for virtual screening. **(F)** Schematic diagram illustrating the five-pocket screening design. For each pocket, the top 200 compounds were retained after docking for downstream cross-pocket comparison and consensus prioritization. **(G)** Docking score distributions of the top-ranked compounds obtained from each pocket. The five pockets showed distinct score profiles, with Pocket-4 exhibiting overall lower docking scores than the other pockets, indicating differences in ligand preference and binding characteristics among the predicted pockets.

We then examined the three-dimensional structure of MPXV TMK and identified five major predicted ligandable pockets on the protein surface (**Figure 3E**). These pockets differ in spatial location, geometric features, and surrounding residue environments, suggesting that TMK may accommodate structurally distinct ligands through multiple binding modes. Based on this observation, the screening strategy was not restricted to a single canonical site, but instead implemented parallel docking across all five pockets.

For each pocket, the top 200 compounds ranked by docking score were retained for subsequent comparison (**Figure 3F**). The distribution of docking scores varied across pockets (**Figure 3G**). Among them, Pocket-4 consistently showed lower docking scores than the other pockets, while Pocket-1, Pocket-2, Pocket-3, and Pocket-5 exhibited partially overlapping yet distinguishable score ranges. These differences suggest that individual pockets impose distinct constraints on ligand binding and may preferentially accommodate different subsets of compounds. Rather than selecting candidates from a single pocket, the top-ranked compounds from all five pockets were carried forward for cross-pocket comparison and integrated prioritization. This strategy reduces the bias associated with single-site screening and allows the identification of molecules with more robust binding potential across different structural contexts.

### 3.4 Cross-pocket consensus prioritization and ADMET evaluation of candidate compounds

Based on the independent docking results obtained from the five predicted pockets, we further performed cross-pocket integration of the top-ranked compounds to identify candidates that consistently performed well across multiple pockets. First, the rank-score profiles of the top 200 compounds from each pocket showed clear differences in docking score distributions among the five pockets, with Pocket-4 exhibiting the lowest overall scores, whereas Pocket-1, Pocket-2, Pocket-3, and Pocket-5 showed relatively similar score ranges (**Figure 4A**). These findings suggest that different pockets possess distinct ligand preferences, and that evaluating compounds solely on the basis of the best score from a single pocket is insufficient to comprehensively assess their binding potential.

**Figure 4:**
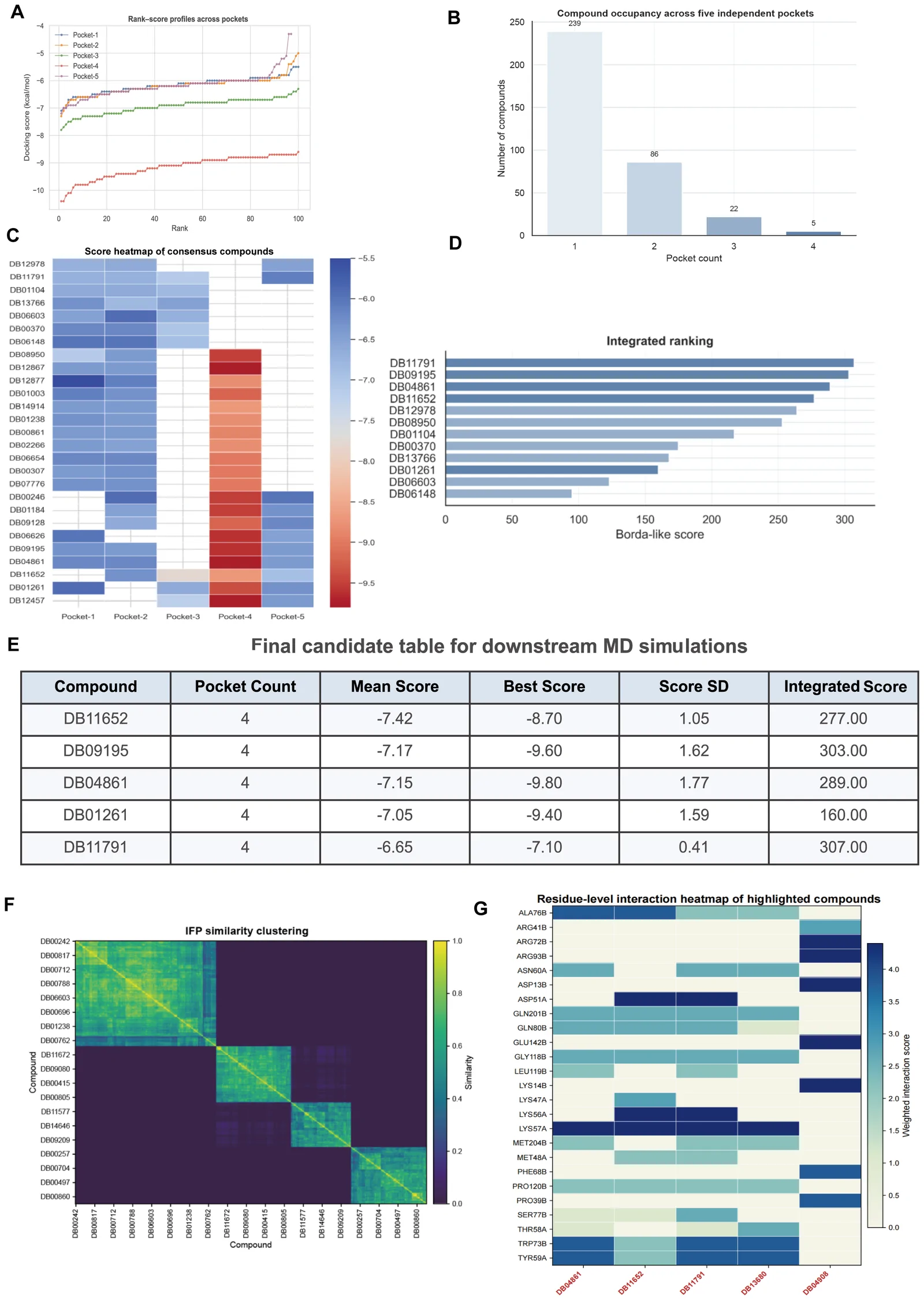
Multi-pocket consensus screening and integrated prioritization of candidate compounds targeting Mpox TMK. **(A)** Rank–score profiles of the top 200 docked compounds across the five predicted pockets of Mpox TMK. The docking score distributions differed among pockets, with Pocket-4 showing overall lower scores than the other four pockets. **(B)** Distribution of compound occupancy across independent pockets. Most candidates were identified in only one pocket, whereas the numbers of compounds simultaneously appearing in 2, 3, and 4 pockets decreased markedly, highlighting the selectivity of cross-pocket consensus screening. **(C)** Heatmap showing the docking score patterns of consensus compounds across Pocket-1 to Pocket-5. Distinct pocket-dependent scoring profiles were observed, indicating different pocket preferences and potential binding modes among the candidate compounds. **(D)** Integrated ranking of consensus candidates based on a Borda-like scoring strategy that incorporated pocket coverage, mean docking score, best docking score, and score variability. The top-ranked compounds were prioritized for further analysis. **(E)** Summary table of candidate compounds identified in four pockets, including pocket count, mean docking score, best docking score, score standard deviation, and integrated rank. **(F)** Interaction fingerprint (IFP) similarity clustering of highlighted compounds. The clustering map shows the global similarity of residue-level interaction patterns among candidate ligands and supports the presence of distinct binding-mode clusters. **(G)** Residue-level interaction heatmap of highlighted compounds. The heatmap summarizes the interaction patterns of candidate compounds with key TMK residues, revealing shared and compound-specific contact patterns that support the structural plausibility of the prioritized hits.

We next analyzed the recurrence of compounds across different pockets and found that most candidates were identified in only one pocket, whereas the numbers of compounds simultaneously appearing in 2, 3, and 4 pockets decreased markedly (**Figure 4B**). This result indicates that compounds repeatedly identified across multiple pockets were relatively rare, and that multi-pocket consensus screening can effectively further narrow down a more robust set of candidates.

Heatmap analysis of the docking scores of consensus compounds across different pockets further revealed substantial differences in their scoring patterns among the five pockets (**Figure 4C**), suggesting that these molecules may possess distinct pocket preferences and binding modes. On this basis, we performed integrated ranking of the consensus candidates by jointly considering pocket coverage, mean docking score, best docking score, and score variability (**Figure 4D**). The results showed that DB11791, DB09195, DB04861, DB11652, and DB12978 ranked among the top candidates. Further analysis of compounds simultaneously identified in four pockets demonstrated that DB11652, DB09195, DB04861, DB01261, and DB11791 all exhibited strong cross-pocket coverage (**Figure 4E**).

To provide additional support for the selection of candidates for subsequent molecular dynamics analysis, we summarized the key ADMET properties of the top five FDA-approved compounds in **Supplementary Table 2**. These ADMET profiles were used only for context-specific prioritization rather than for re-assessing the clinical safety of approved drugs. Although all candidates exhibited favorable predicted intestinal absorption, they differed substantially in BBB penetration, efflux tendency, microsomal stability, and predicted safety-related signals. By contrast, DB01261 and DB11652 showed less favorable predicted ADMET profiles. Overall, these ADMET results provided supplementary evidence for context-specific prioritization of the candidates in the antiviral repurposing framework.

To further evaluate the binding plausibility of these candidates, we performed clustering analysis of their interaction fingerprints. The results showed that the top-ranked candidates formed relatively clear clusters in terms of interaction patterns (**Figure 4F**). In addition, the residue-level interaction heatmap further demonstrated that these compounds shared relatively consistent contact patterns with multiple key residues (**Figure 4G**). Under this integrated framework, DB11791, DB04861, and DB11652 were prioritized for subsequent molecular dynamics simulations because they showed favorable overall performance across multiple evaluation layers, including cross-pocket recurrence, integrated docking ranking, interaction-pattern plausibility, and comparative ADMET profiles. These compounds were therefore selected as representative candidates for dynamic validation.

### 3.5 Molecular dynamics simulations prioritize DB11791 as a comparatively stable TMK-binding candidate

To further assess the dynamic binding stability of the prioritized compounds, three independent 150 ns MD simulations were carried out for DB04861, DB11652, and DB11791, with the co-crystallized ligand TYD serving as the reference. The representative initial binding conformations of the four TMK-ligand complexes are presented in **Figure 5A**.

**Figure 5:**
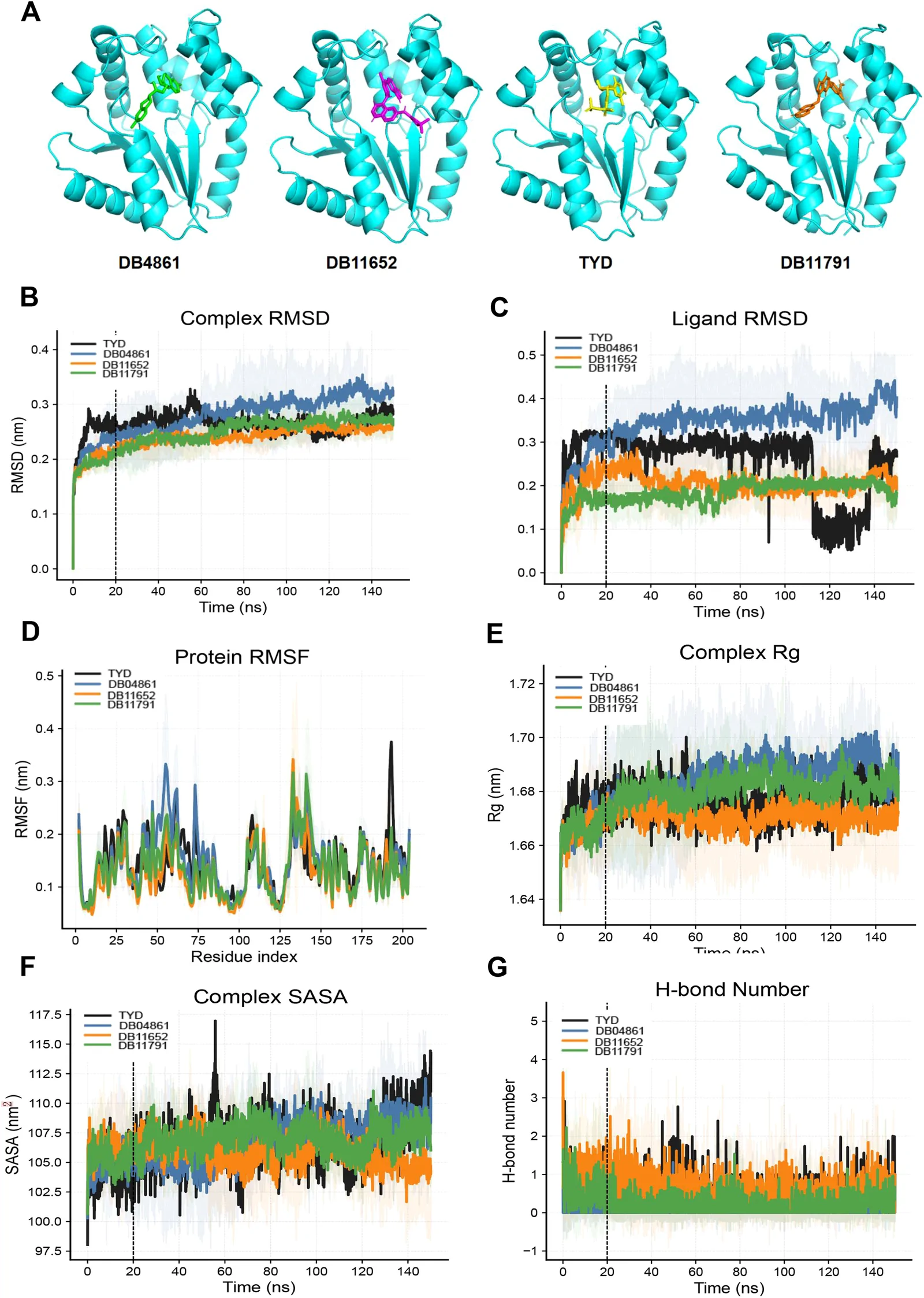
Molecular dynamics comparison of prioritized compounds bound to Mpox TMK. **(A)** Representative initial binding conformations of DB04861, DB11652, TYD, and DB11791 in the TMK binding pocket. TMK is shown in cyan cartoon representation, and ligands are shown as sticks in different colors. **(B)** Complex RMSD trajectories of the four TMK–ligand systems during 150 ns molecular dynamics simulations. **(C)** Ligand RMSD trajectories showing the positional stability of each ligand within the binding pocket. **(D)** Protein RMSF profiles of TMK in complex with TYD, DB04861, DB11652, and DB11791. **(E)** Radius of gyration (Rg) of the four TMK–ligand complexes during the simulation. **(F)** Solvent-accessible surface area (SASA) of the four TMK–ligand complexes. **(G)** Hydrogen-bond number between TMK and each ligand during the simulation. In panels **(B, C, E-G)** the dashed vertical line indicates the approximate transition from the initial equilibration phase to the subsequent relatively stable sampling stage.

Complex RMSD trajectories showed that all four systems underwent an initial equilibration period followed by a gradual transition into relatively stable conformational states, indicating that each ligand was able to maintain a stable association with TMK during the simulation (**Figure 5B**). Among these systems, the DB04861-bound complex displayed larger fluctuations and a higher RMSD in the later stage, whereas the DB11652- and DB11791-bound complexes remained comparatively steady throughout the trajectory.

Ligand RMSD provided a more direct measure of ligand retention within the binding pocket and revealed clearer differences among the tested compounds (**Figure 5C**). DB04861 exhibited the most evident positional drift, consistent with weaker pocket retention. DB11652 showed moderate ligand stability. By contrast, DB11791 maintained the lowest and most stable ligand RMSD across the simulation, indicating minimal conformational displacement and stronger retention within the binding site than the other candidate compounds. Although TYD remained generally stable, its fluctuation amplitude was still greater than that of DB11791.

Protein RMSF profiles further showed that the overall residue-level flexibility patterns were similar across the four systems, with fluctuations mainly confined to several local regions rather than distributed across the entire protein structure (**Figure 5D**). In the DB11791-bound system, no apparent increase in global residue mobility was observed, supporting the structural integrity of the TMK complex during the simulation.

Analysis of the radius of gyration (Rg) showed that all systems experienced a modest conformational adjustment at the beginning of the simulation and then fluctuated within a relatively narrow range, suggesting that ligand binding did not induce obvious global loosening or collapse of the protein structure (**Figure 5E**). Among the candidate compounds, DB11791 and DB11652 displayed more stable Rg profiles during the middle and late stages of the trajectory.

The SASA results were in line with the Rg analysis. All complexes fluctuated within a similar range during the simulation, with no clear sign of abnormal solvent exposure or structural disruption (**Figure 5F**). These data support that binding of the candidate ligands did not markedly destabilize the overall TMK fold.

Hydrogen-bond analysis showed that ligand-associated hydrogen-bond interactions were maintained in all four systems, although the dynamic patterns differed among them (**Figure 5G**). TYD generally displayed a higher but more variable hydrogen-bond number, whereas the three candidate compounds showed lower yet more stable hydrogen-bond profiles. This pattern suggests that the stability of these candidates may rely less on persistent hydrogen bonding alone and more on the combined contribution of multiple non-covalent interactions.

Differences among the three prioritized compounds became most evident at the ligand level. DB11791 showed the smallest ligand drift, stable complex maintenance, and consistent pocket retention throughout the simulation, and was therefore selected for subsequent in-depth conformational and energetic analyses.

### 3.6 In-depth conformational and energetic analyses further support DB11791 as a prioritized computational candidate

Building on the MD results shown in **Figure 5**, we further examined DB11791 at the conformational and energetic levels to clarify its advantage over the other candidates and the reference ligand TYD.

Pocket-retention analysis showed that DB11791 consistently had the highest fraction of stable frames across different ligand RMSD thresholds, followed by DB11652, whereas DB04861 showed the weakest retention (**Figure 6A**). This indicates that DB11791 maintained the most persistent binding pose within the TMK pocket.

**Figure 6:**
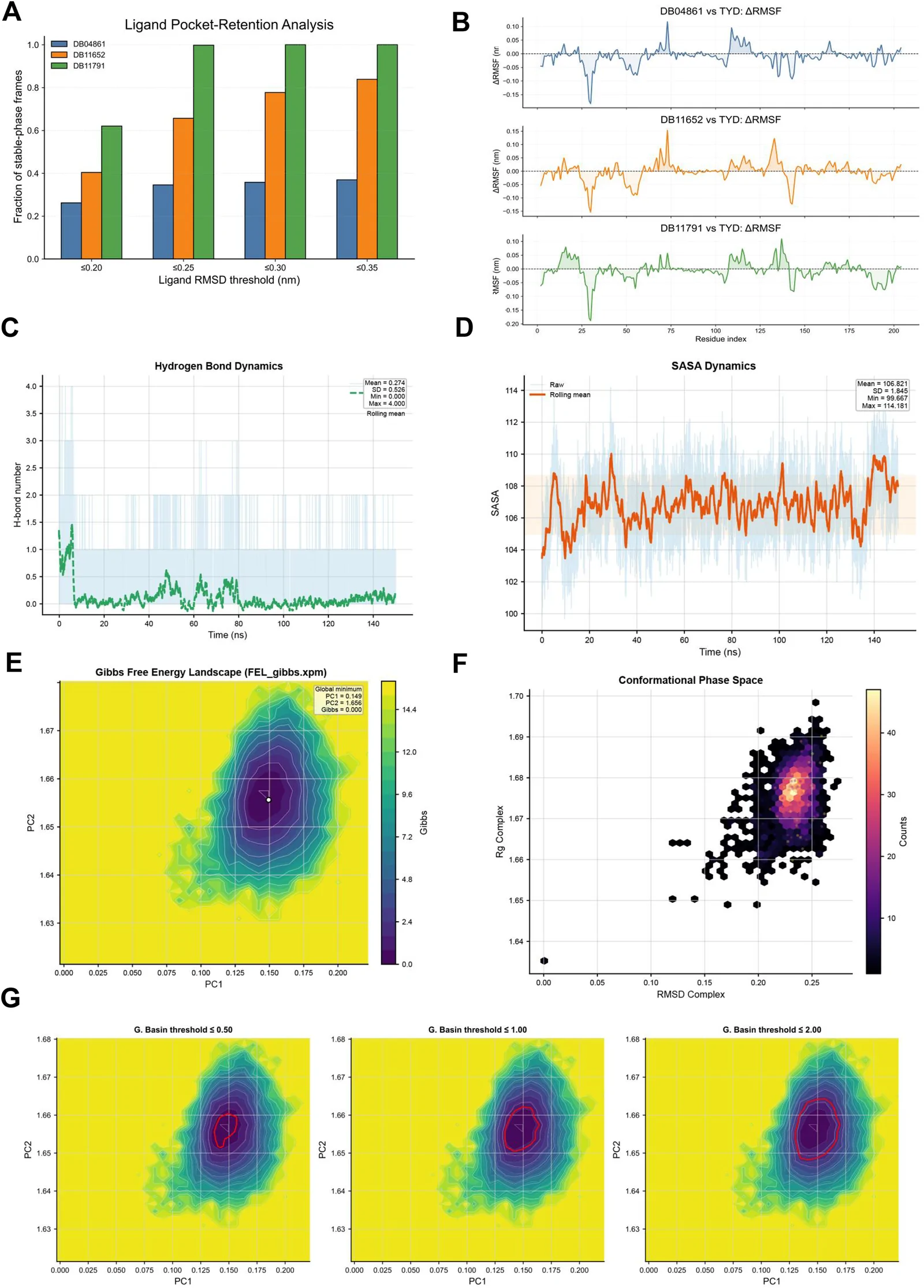
In-depth conformational and energetic analyses of DB11791 bound to Mpox TMK. **(A)** Ligand pocket-retention analysis of DB04861, DB11652, and DB11791 under different ligand RMSD thresholds, showing the fraction of stable frames maintained within the TMK binding pocket. **(B)** Residue-level ΔRMSF profiles of DB04861-, DB11652-, and DB11791-bound TMK complexes relative to the TYD-bound reference system. **(C)** Hydrogen-bond dynamics of the DB11791–TMK complex during the molecular dynamics simulation. The light blue bars indicate instantaneous hydrogen-bond counts, and the green curve represents the smoothed trend. **(D)** SASA dynamics of the DB11791–TMK complex. The light blue trace indicates the raw SASA values, and the orange curve represents the rolling mean. **(E)** Gibbs free energy landscape of the DB11791–TMK complex constructed from the first two principal components (PC1 and PC2). The white point marks the global minimum. **(F)** Conformational phase-space analysis of the DB11791–TMK complex, showing the distribution of sampled conformations in the RMSD–Rg space. **(G)** Basin contour analysis of the DB11791–TMK complex under Gibbs free energy thresholds of ≤ 0.50, ≤ 1.00, and ≤ 2.00, respectively. Red contours indicate the dominant low-energy basin under each threshold.

Compared with the TYD-bound system, all three candidate compounds altered local residue fluctuations to some extent, but DB11791 caused only limited overall deviation, with no sign of widespread abnormal flexibility (**Figure 6B**). This suggests that DB11791 preserved the global structural stability of TMK while allowing modest local adjustment.

In the DB11791-bound system, hydrogen-bond interactions were relatively abundant in the early stage of the simulation, then gradually decreased and stabilized at a lower level in the later stage (**Figure 6C**). This pattern is consistent with an initial accommodation process followed by a more equilibrated binding state. The SASA profile showed a similar trend, remaining within a stable range throughout the trajectory with only a slight increase at the later stage (**Figure 6D**), indicating that DB11791 binding did not induce abnormal solvent exposure or obvious destabilization of TMK.

The Gibbs free energy landscape based on the first two principal components showed that the DB11791-TMK complex mainly converged into a relatively concentrated low-energy basin with a continuous distribution along the diagonal direction (**Figure 6E**). Consistently, phase-space analysis showed that the major sampled conformations were confined to a limited region and formed relatively clear state clusters (**Figure 6F**), supporting constrained and reproducible conformational sampling.

Under basin thresholds of ≤ 0.50, ≤ 1.00, and ≤ 2.00, the DB11791-bound system consistently formed continuous and compact contours around the same principal low-energy region (**Figure 6G**). Although the basin area expanded with increasing threshold, the core low-energy center remained essentially unchanged, indicating a stable dominant conformational state rather than multiple separated metastable states.

Collectively, these analyses further support DB11791 as the strongest candidate, with better conformational convergence and a more clearly defined dominant low-energy state than the other compounds examined.

## 4 Discussion

The continued global concern surrounding mpox has highlighted the need for new antiviral strategies beyond currently available therapeutic options. Although several approved agents have been considered for orthopoxvirus infection, target-specific small-molecule discovery against essential viral enzymes remains limited. In this study, we focused on MPXV TMK as a potential antiviral target and developed a structure-guided, multi-pocket virtual screening workflow to identify and prioritize candidate compounds for further evaluation. By integrating compound library characterization, five-pocket docking, cross-pocket consensus prioritization, interaction fingerprint analysis, and molecular dynamics simulations, we ultimately identified DB11791 as the computationally prioritized candidate.

TMK represents an attractive antiviral target because of its central role in viral nucleotide metabolism and DNA replication. Viral thymidylate kinase catalyzes the phosphorylation of thymidine monophosphate and contributes to the nucleotide supply required for efficient viral genome synthesis. From a drug discovery perspective, enzymes involved in nucleotide metabolism are particularly appealing because they are functionally indispensable and structurally tractable. Our structural and sequence analyses further supported the relevance of TMK by showing that it contains conserved functional regions while still presenting multiple ligandable pockets, thereby offering opportunities for both canonical and non-canonical inhibitor binding. This provided the basis for exploring TMK not simply as a single-site docking target, but as a protein with a broader and potentially more complex inhibitor-binding landscape.

A major feature of the present study is the use of a multi-pocket consensus screening strategy. Conventional structure-based screening often relies on a single active site, which may bias compound selection toward molecules optimized for one local geometry while overlooking ligands capable of binding alternative or partially overlapping pockets. In contrast, our analysis identified five major putative pockets on MPXV TMK and showed that these pockets differed substantially in docking score distribution and ligand preference. Notably, most compounds appeared only in one pocket, whereas compounds repeatedly identified across multiple pockets were relatively rare. This finding suggests that cross-pocket recurrence itself provides useful discriminative information. Therefore, rather than prioritizing compounds solely on the basis of the best docking score from a single pocket, we integrated pocket coverage, mean score, best score, and score variability to identify more robust candidates. This strategy likely reduced pocket-specific bias and improved the reliability of downstream prioritization.

Another important aspect of our workflow is that compound selection was not based on docking score alone. The top consensus candidates were further evaluated using interaction fingerprint clustering and residue-level interaction analysis. These complementary analyses helped determine whether high-ranking molecules also shared plausible and coherent interaction patterns with the protein. This is particularly important in virtual screening studies, where score-based prioritization alone may enrich false positives. In our results, the highlighted candidates not only showed favorable integrated ranking but also formed relatively consistent contact patterns with multiple key residues, suggesting that their binding poses were structurally reasonable rather than purely score-driven artifacts.

Among the prioritized compounds, DB11791 emerged as the most consistently supported computational candidate. Although all three selected compounds-DB04861, DB11652, and DB11791-were capable of forming dynamically stable complexes with MPXV TMK, their ligand-level behaviors differed substantially. Complex RMSD, Rg, and SASA analyses indicated that all systems reached relatively stable conformational regimes after the initial equilibration stage, suggesting that none of the candidates caused obvious destabilization of the protein. However, ligand RMSD and pocket-retention analyses revealed clearer differences. DB04861 exhibited more pronounced positional drift, whereas DB11652 showed intermediate stability. In contrast, DB11791 consistently maintained the lowest ligand RMSD and the highest fraction of stable frames across different thresholds, indicating comparatively greater binding-pose retention within the pocket under the simulated conditions. This distinction is important because global complex stability does not necessarily guarantee stable ligand accommodation, whereas ligand-level stability more directly reflects the likelihood of persistent target engagement.

The in-depth conformational and energetic analyses further strengthened the case for DB11791. Relative to TYD, DB11791 induced only limited local residue flexibility changes and did not trigger large-scale abnormal fluctuations, suggesting that it was well accommodated within the TMK structure while preserving global conformational integrity. Hydrogen-bond and SASA analyses suggested that the DB11791-bound complex underwent an initial accommodation phase followed by stabilization into a more persistent binding state. More importantly, Gibbs free energy landscape analysis showed that the DB11791-TMK complex converged into a relatively concentrated low-energy basin rather than dispersing broadly across multiple conformational states. Conformational phase-space analysis and basin contour quantification under multiple thresholds further indicated that this dominant low-energy region remained stable and continuous. Together, these observations suggest that DB11791 is associated with a more favorable and better-converged conformational ensemble, further supporting its favorable dynamic profile among the tested compounds.

From a broader perspective, our findings support the value of combining docking-based prioritization with dynamic and energy-landscape-based validation. In many antiviral repurposing or screening studies, candidate selection ends at static docking poses or simple score ranking. However, viral proteins often undergo local rearrangements upon ligand binding, and compounds with apparently favorable static scores may not remain stable during simulation. Our results illustrate this point clearly: although DB04861 performed well in some docking-based metrics, it showed more evident ligand drift during MD simulation. By contrast, DB11791 showed a more balanced profile across docking, interaction patterning, dynamic stability, and conformational convergence. This underscores the importance of using a layered strategy in antiviral structure-based discovery.

Despite these strengths, the present study has several limitations. First, the screening and prioritization framework is computational and predictive in nature. Although our docking, interaction fingerprinting, and MD analyses consistently prioritize DB11791 as a computational candidate, direct experimental validation is still required to confirm its inhibitory activity against MPXV TMK. Biochemical enzyme inhibition assays, thermal shift assays, and orthogonal binding measurements will be necessary to determine whether the predicted interaction translates into measurable target engagement. Second, although three independent 150 ns MD replicates were performed, broader sampling and longer simulations would further strengthen the robustness of the dynamic and energetic interpretation. Longer simulations, replicate trajectories, and free-energy-based binding affinity calculations such as MM/PBSA, MM/GBSA, or alchemical approaches would further strengthen the energetic interpretation. Third, because TMK belongs to a nucleotide metabolism-related enzyme family, the selectivity of DB11791 against host kinases or related enzymes remains unknown and should be carefully evaluated in future studies.

In addition, the current work does not yet address antiviral efficacy at the cellular or organismal level. Even if DB11791 binds favorably to MPXV TMK in silico, its ultimate therapeutic relevance will depend on additional properties, including solubility, membrane permeability, intracellular exposure, metabolic stability, and safety. Therefore, the next stage of investigation should combine biochemical validation with antiviral assays in relevant cellular infection models, followed by structure-activity relationship optimization if necessary. Although the present multi-pocket docking and molecular dynamics analyses support a preferred TMK-binding region for DB11791, the exact binding mode and residue-level interaction network still require further biochemical and structural validation.

In conclusion, this study establishes a structure-guided multi-pocket consensus screening framework for MPXV TMK and demonstrates that integrating cross-pocket docking, interaction fingerprint analysis, and molecular dynamics simulations can effectively prioritize candidate compounds for further experimental evaluation. Among the screened compounds, DB11791 showed the most favorable overall profile, including strong pocket retention, stable ligand behavior, consistent protein conformational behavior, and convergence toward a dominant low-energy conformational state. These findings prioritize DB11791 as a computational candidate for further biochemical and antiviral validation and provide a practical computational framework for antiviral inhibitor discovery targeting MPXV TMK.

## Data Availability

The original contributions presented in the study are included in the article and its **Supplementary Material**. Further inquiries can be directed to the corresponding author.

## References

[B1] Ahmed S. K. Ahmed Rashad E. A. Mohamed M. G. Ravi R. K. Essa R. A. Abdulqadir S. O. . (2022). The global human monkeypox outbreak in 2022: An overview. Int. J. Surg. 104, 106794. doi:10.1016/j.ijsu.2022.106794 35918003 PMC9624120

[B2] Alakunle E. Moens U. Nchinda G. Okeke M. I. (2020). Monkeypox Virus in Nigeria: Infection Biology, Epidemiology, and Evolution. Viruses 12, 1257. doi:10.3390/v12111257 33167496 PMC7694534

[B3] (2024). Global strategic preparedness and response plan launched by WHO to contain mpox outbreak. Saudi Med. J. 45, 1202–1203. doi:10.15537/1658-3175.8467 39379123 PMC11463572

[B4] Black E. J. Powell C. S. Dempsey D. M. Hendrickson R. C. Mims L. R. Lefkowitz E. J. (2026). Virus taxonomy: the database of the International Committee on Taxonomy of Viruses. Nucleic Acids Res. 54, D776–D789. doi:10.1093/nar/gkaf1159 41296552 PMC12807731

[B5] Breman J. G. Kalisa-Ruti Steniowski M. V. Zanotto E. Gromyko A. I. Arita I. (1980). Human monkeypox, 1970-79. Bull. World Health Organ. 58, 165–182. PMC2395797 6249508

[B6] Caillat C. Topalis D. Agrofoglio L. A. . (2008). Crystal structure of poxvirus thymidylate kinase: an unexpected dimerization has implications for antiviral therapy. Proc. Natl. Acad. Sci. U.S.A. 105, 16900–16905. doi:10.1073/pnas.0804525105 18971333 PMC2579350

[B7] Falendysz E. A. Lopera J. G. Doty J. B. Nakazawa Y. Crill C. Lorenzsonn F. . (2017). Characterization of monkeypox virus infection in African rope squirrels (Funisciurus sp.). PLoS Negl. Trop. Dis. 11, e0005809. doi: 10.1371/journal.pntd.0005809 PMC5578676 28827792

[B8] Ferrareze P. A. G. Pereira E Costa R. A. Thompson C. E. (2023). Genomic characterization and molecular evolution of human monkeypox viruses. Arch. Virol. 168, 278. doi:10.1007/s00705-023-05904-5 37864757

[B9] Gan J. H. Liu J. X. Liu Y. Chen S. W. Dai W. T. Xiao Z. X. . (2023). DrugRep: an automatic virtual screening server for drug repurposing. Acta Pharmacol. Sin. 44, 888–896. doi:10.1038/s41401-022-00996-2 36216900 PMC9549438

[B10] Gasteiger J. Marsili M. (1980). Iterative partial equalization of orbital electronegativity-a rapid access to atomic charges. Tetrahedron 36, 3219–3228. doi:10.1016/0040-4020(80)80168-2

[B11] Gessain A. Nakoune E. Yazdanpanah Y. (2022). Monkeypox. N. Engl. J. Med. 387, 1783–1793. doi:10.1056/nejmra2208860 36286263

[B12] Gigante C. M. Korber B. Seabolt M. H. . (2022). Multiple lineages of MPXV detected in the United States, 2021-2022. Science 378, 560–565. doi:10.1126/science.add4153 36264825 PMC10258808

[B13] Gu Y. Yu Z. Wang Y. . (2024). admetSAR3.0: a comprehensive platform for exploration, prediction and optimization of chemical ADMET properties. Nucleic Acids Res. 52, W432–W438. doi:10.1093/nar/gkae298 38647076 PMC11223829

[B14] Hraib M. Jouni S. Albitar M. M. . (2022). The outbreak of monkeypox 2022: An overview. Ann. Med. Surg. (Lond) 79, 104069. doi:10.1016/j.amsu.2022.104069 35860140 PMC9289401

[B15] Hutson C. L. Nakazawa Y. J. Self J. Olson V. A. Regnery R. L. Braden Z. . (2015). Laboratory investigations of African pouched rats (Cricetomys gambianus) as a potential reservoir host species for monkeypox virus. PLoS Negl. Trop. Dis. 9, e0004013. doi: 10.1371/journal.pntd.0004013 PMC4627651 26517724

[B16] Isidro J. Borges V. Pinto M. . (2022). Phylogenomic characterization and signs of microevolution in the 2022 multi-country outbreak of MPXV. Nat. Med. 28, 1569–1572. doi:10.1038/s41591-022-01907-y 35750157 PMC9388373

[B17] Laurenson-Schafer H. Sklenovská N. Hoxha A. Kerr S. M. Ndumbi P. Fitzner J. . (2023). Description of the first global outbreak of mpox: an analysis of global surveillance data. Lancet Glob. Health 11, e1012–e1023. doi:10.1016/s2214-109x(23)00198-5 37349031 PMC10281644

[B18] Lemoine F. Correia D. Lefort V. Doppelt-Azeroual O. Mareuil F. Cohen-Boulakia S. . (2019). NGPhylogeny.fr: new generation phylogenetic services for non-specialists. Nucleic Acids Res. 47, W260–W265. doi: 10.1093/nar/gkz303 PMC6602494 31028399

[B19] Li Y. Shen Y. Hu Z. Yan R. (2023). Structural basis for the assembly of the DNA polymerase holoenzyme from a monkeypox virus variant. Sci. Adv. 9, eadg2331. doi:10.1126/sciadv.adg2331 37075110 PMC10115419

[B20] Liu Y. Grimm M. Dai W. T. . (2020). CB-Dock: a web server for cavity detection-guided protein-ligand blind docking. Acta Pharmacol. Sin. 41, 138–144. doi:10.1038/s41401-019-0228-6 31263275 PMC7471403

[B21] Luna N. Ramírez A. L. Muñoz M. Ballesteros N. Patiño L. H. Castañeda S. A. . (2022). Phylogenomic analysis of the monkeypox virus (MPXV) 2022 outbreak: Emergence of a novel viral lineage? Travel Med. Infect. Dis. 49, 102402. doi:10.1016/j.tmaid.2022.102402 35840078 PMC9628808

[B22] Manes N. P. Estep R. D. Mottaz H. M. Moore R. J. Clauss T. R. Monroe M. E. . (2008). Comparative proteomics of human monkeypox and vaccinia intracellular mature and extracellular enveloped virions. J. Proteome Res. 7, 960–968. doi:10.1021/pr070432+ 18205298 PMC2517256

[B23] McInnes C. J. Lefkowitz E. J. . (2023). ICTV Virus Taxonomy Profile: Poxviridae 2023. J. Gen. Virol. 104, 1849. doi:10.1099/jgv.0.001849 37195882 PMC12447285

[B24] McQuiston J. H. Braden C. R. Bowen M. D. . (2023). The CDC Domestic Mpox Response—United States, 2022–2023. MMWR Morb Mortal Wkly Rep. 72, 547–552. doi:10.15585/mmwr.mm7220a2 37200231 PMC10205168

[B25] Minasov G. Inniss N. L. Shuvalova L. Anderson W. F. Satchell K. J. F. (2022). Structure of the Monkeypox virus profilin-like protein A42R reveals potential functional differences from cellular profilins. Acta Crystallogr. F Struct. Biol. Commun. 78, 371–377. doi:10.1107/s2053230x22009128 36189721 PMC9527652

[B26] Morris G. M. Huey R. Lindstrom W. . (2009). AutoDock4 and AutoDockTools4: Automated docking with selective receptor flexibility. J. Comput. Chem. 30, 2785–2791. doi:10.1002/jcc.21256 19399780 PMC2760638

[B27] Moss B. (2013). Poxvirus DNA replication. Cold Spring Harb. Perspect. Biol. 5, a010199. doi:10.1101/cshperspect.a010199 23838441 PMC3753712

[B28] O’Boyle N. M. Banck M. James C. A. . (2011). Open Babel: An open chemical toolbox. J. Cheminform 3, 33. doi: 10.1186/1758-2946-3-33 PMC3198950 21982300

[B29] Prichard M. N. Kern E. R. (2012). Orthopoxvirus targets for the development of new antiviral agents. Antiviral Res. 94, 111–125. doi:10.1016/j.antiviral.2012.02.012 22406470 PMC3773844

[B30] Resch W. Hixson K. K. Moore R. J. Lipton M. S. Moss B. (2007). Protein composition of the vaccinia virus mature virion. Virology 358, 233–247. doi:10.1016/j.virol.2006.08.025 17005230

[B31] Riutord-Fe C. Schlotterbeck J. Lagostina L. Kouadio L. Herridge H. R. Jochum M. J. S. . (2026). Transmission of MPXV from fire-footed rope squirrels to sooty mangabeys. Nature 651, 185–190. doi: 10.1038/s41586-025-10086-y PMC12960232 41673146

[B32] Robert X. Gouet P. (2014). Deciphering key features in protein structures with the new ENDscript server. Nucleic Acids Res. 42, W320–W324. doi: 10.1093/nar/gku316 PMC4086106 24753421

[B33] Schmidt F. I. Bleck C. K. Mercer J. (2012). Poxvirus host cell entry. Curr. Opin. Virol. 2, 20–27. doi:10.1016/j.coviro.2011.11.007 22440962

[B34] Shchelkunov S. N. Totmenin A. V. Babkin I. V. Safronov P. F. Ryazankina O. I. Petrov N. A. . (2001). Human monkeypox and smallpox viruses: genomic comparison. FEBS Lett. 509, 66–70. doi:10.1016/s0014-5793(01)03144-1 11734207 PMC9533818

[B35] Siami H. Asghari A. Parsamanesh N. (2023). Monkeypox: Virology, laboratory diagnosis and therapeutic approach. J. Gene Med. 25, e3521. doi:10.1002/jgm.3521 37132057

[B36] Tang H. Zhang A. (2023). Human mpox: Biology, epidemiology, therapeutic options, and development of small molecule inhibitors. Med. Res. Rev. 43, 1019–1037. doi:10.1002/med.21943 36891882

[B37] Trott O. Olson A. J. (2010). AutoDock Vina: improving the speed and accuracy of docking with a new scoring function, efficient optimization, and multithreading. J. Comput. Chem. 31, 455–461. doi:10.1002/jcc.21334 19499576 PMC3041641

[B38] Von Magnus P. Andersen E. K. Petersen K. B. Birch-Andersen A. (1959). A pox-like disease in cynomolgus monkeys. Acta Pathologica Microbiologica Scandinavica 46, 156–176. doi:10.1111/j.1699-0463.1959.tb00328.x41940437

[B39] Wishart D. S. Feunang Y. D. Guo A. C. . (2018). DrugBank 5.0: a major update to the DrugBank database for 2018. Nucleic Acids Res. 46, D1074–D1082. doi:10.1093/nar/gkx1037 29126136 PMC5753335

[B40] World Health Organization (2022). “ WHO Director-General declares the ongoing monkeypox outbreak a Public Health Emergency of International Concern,” in World Health Organization ( World Health Organization, Geneva). Internet.

